# Maternal SARS‐CoV‐2 infection: The potential vertical transmission of SARS‐CoV‐2 and impact on neonates: A review

**DOI:** 10.1002/pdi3.22

**Published:** 2023-08-22

**Authors:** Wei Xia, Zhoujie Peng

**Affiliations:** ^1^ Department of Neonatology Chongqing University Three Gorges Hospital Chongqing China

**Keywords:** COVID‐19, neonates, perinatal transmission, SARS‐CoV‐2, vertical transmission

## Abstract

Severe acute respiratory syndrome coronavirus 2 (SARS‐CoV‐2), a large, lipid‐enveloped, single‐stranded RNA virus, is a highly contagious virus that caused coronavirus disease 2019 (COVID‐19), declared a pandemic by the World Health Organization on March 11, 2020. Pregnant women are usually considered at high risk for infectious diseases, including COVID‐19. Maternal SARS‐CoV‐2 infection can adversely affect the pregnancy and birth outcomes, such as abortion, intrauterine growth restriction, and prematurity. Some meta‐analysis suggested that the outcomes of newborns are different between symptomatic and asymptomatic pregnant women but similar in asymptomatic and SARS‐CoV‐2 negative group. Maternal infection increases the risk of vertical transmission; also the presence of SARS‐CoV‐2 or its RNA in maternal samples in some case reports raised the possibility of intrauterine transmission. Also, contact transmission during delivery and postnatal transmission are discussed. Although most infected newborns are asymptomatic or mildly symptomatic, there are case reports of severe neonatal SARS‐CoV‐2 infection, including cardiorespiratory failure and death. Otherwise, some studies suggested that the COVID‐19 pandemic was associated with a reduction for preterm birth during the pandemic compared with the prepandemic period. We conduct this review to try to make a conclusion about the vertical transmission of SARS‐CoV‐2 and impact on neonates due to Maternal SARS‐CoV‐2 infection.

## 1 INTRODUCTION

Severe acute respiratory syndrome coronavirus 2 (SARS‐CoV‐2), a large, lipid‐enveloped, single‐stranded RNA virus, is a highly contagious virus that causes coronavirus disease 2019 (COVID‐19). SARS‐CoV‐2 spread across China in December 2019.^1^ Moreover, the reported cases in other countries also increased rapidly, and the virus was declared a pandemic by the World Health Organization (WHO) on March 11, 2020.^2^ The SARS‐CoV‐2 B.1.617 lineage, that is, the Delta strain, gained global attention since March 2021 and was identified in October 2020 in India. Since then, it has been the dominant strain in some regions and has spread to other countries.^3^ B.1.1.529 was first detected in Botswana and on November 14, 2021, in South Africa. It was first reported to the WHO on November 24. On November 26, the WHO defined it as the fifth variant of concern (named it Omicron).^4^ Pregnant women are usually considered at high risk for infectious diseases due to immunologic, physiologic, and cardiopulmonary changes in their bodies during pregnancy.^5^, ^6^ Thus, as the uterus expands, the diaphragm is pushed to a higher position; this can create an obstacle for the lungs to expand.^7^ The intolerance to hypoxemia makes pregnant women more likely to develop respiratory disease complications.^8^, ^9^ Maternal severe acute SARS‐CoV‐2 infection can adversely affect the pregnancy and birth outcomes. Fetal complications include abortion, intrauterine growth restriction, and prematurity.^10^ Besides, SARS‐CoV‐2 infection in neonatal population can lead to hypoxia, respiratory distress, vomiting, cough, and fever.^11^ Neonates would present more severe illness than older babies too.^1^, ^11^ Mother‐to‐child transmission of SARS‐CoV‐2 has been proved to exist but the probable routes are unclear. Whether intrauterine transmission is confirmed? Can SARS‐CoV‐2 transmit Mother‐to‐child by breast feeding or vaginal delivery? So, we conduct this review to try to summarize the vertical transmission of SARS‐CoV‐2 and neonates outcome due to Maternal SARS‐CoV‐2 infection.

## 2 PATHOBIOLOGY OF COVID‐19 DURING PREGNANCY AT MATERNAL‐FETAL INTERFACE

As we know, when infected with virus, there are some cytokines produced by T‐helper (TH) lymphocytes regulating immune and inflammation response. The TH1‐type cytokines are pro‐inflammatory mediators, TH2‐type cytokines are anti‐inflammatory factors, conversely.^12^ During pregnancy, the anti‐inflammatory TH2 is the dominant immune response mode compared to pro‐inflammatory TH‐1 to protect the fetus. This shift in the inflammatory cell increases maternal susceptibility to viral pathogens such as SARS‐CoV‐2. The placenta functions as a physiological and immunological barrier to prevent the mother‐to‐fetus viral transfer. A study find that compared to control group, the placentas from patients with SARS‐CoV‐2 were significantly more likely to show abnormal or injured maternal vessels and intervillous thrombi.^13^ SARS‐CoV‐2 infects host respiratory epithelial cells through angiotensin converting enzyme 2 (ACE2). The placental syncytiotrophoblast, cytotrophoblasts, and the extra‐villous trophoblasts express the ACE2 receptors which are also necessary for S protein processing, viral replication, and budding.^14^, ^15^ The maternal side was red to brown with locally small yellow to gray firm areas, diffuse small hemorrhages, and congested blood vessels.^16^ The whole of placenta presents diffuse necrosis of cyto‐ and syncytiotrophoblast, accompanied by perivillous fibrin deposition. Chorionic villi were almost all avascular, focally necrotic.^16^ There was an intense mixed cellular predominantly neutrophilic infiltrate with karyorrhexis in the basal decidua that was spreading into the intervillous space.^17^ Although viral infections during pregnancy are common transplacental passage, fetal infection appears to be rare.^18^ Systematic meta‐analysis did not find increased stillbirth risk during the early months of the SARS‐CoV‐2 pandemic,^19^ but increased rates have been reported during the Delta pandemic.^20^, ^21^ SARS‐CoV‐2 placentitis with thrombohematomas seems a result of severe viral placental damage related to increased stillbirth risk in pregnancy.^22^

## 3 POTENTIAL VERTICAL TRANSMISSION OF SARS‐CoV‐2

**FIGURE 1:**
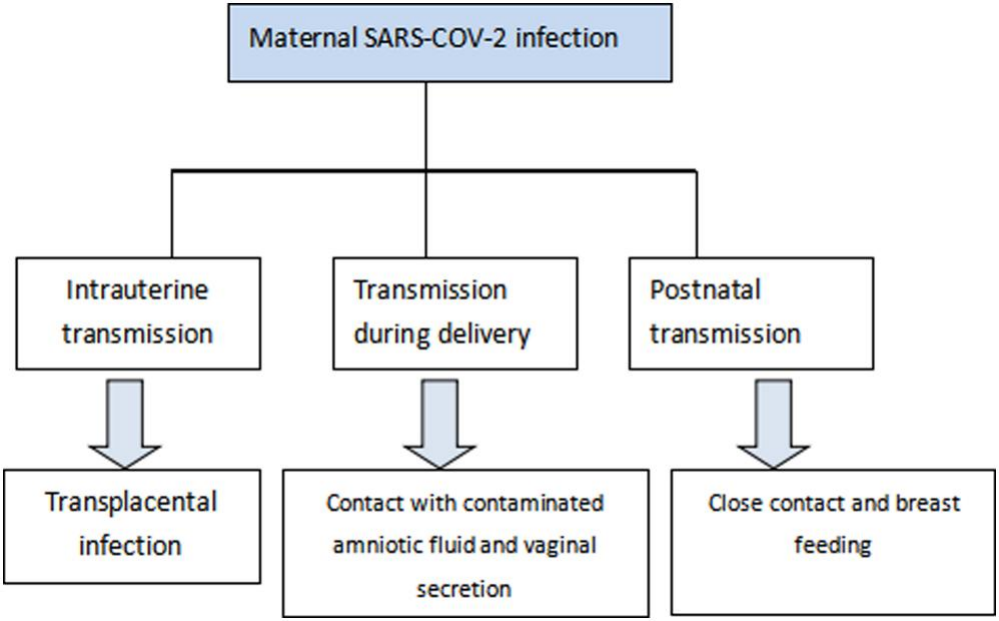
Potential vertical transmission of SARS‐CoV‐2. SARS‐CoV‐2, severe acute respiratory syndrome coronavirus 2.

We consider vertical transmission as baby acquiring infection from its mother. Based on this, we divided the probable routes of SARS‐CoV‐2 transmission (Figure 1) into the following: (1) intrauterine infection via blood or via transmission through swallowed or aspirated amniotic fluid; (2) exposure to maternal blood, vaginal secretions, or feces during delivery; (3) direct contact with the infected mother or other caregivers after delivery, via the respiratory route or through breast milk. So intrauterine transmission (Table 1), contact transmission during delivery (Table 2), and postnatal transmission (Table 3) are discussed here.

**TABLE 1:** Probable intrauterine transmission.

	Mother with SARS‐CoV‐2	Newborn with positive result	Positive samples from newborns
Prospective observational study^23^	209	6/191	RT‐PCR test NP swab
Case report^25^	1	1	Antigen test and RT‐PCR test on a NP swab
Case report^26^	11	3	Placental or membrane swabs
Case report^27^	1	1	Nasopharyngeal swabs positive, multiple areas of infiltration by inflammatory cells
Case report^30^	1	1	SARS‐CoV‐2 N‐protein and Spike RBD were detected in the esophagus, stomach, spleen, and heart

Abbreviation: RT‐PCR, reverse transcription‐polymerase chain reaction.

**TABLE 2:** Contact transmission during delivery.

	Mother with SARS‐CoV‐2	Positive vaginal sample	Positive newborns
A prospective cross‐sectional study^35^	48	2	1 (no positive placental or vaginal samples)
A prospective study^36^	1086	545	8 (no positive rectal or vaginal samples)
A pilot study^37^	50	0	
Observe study^38^	10	0	

**TABLE 3:** Postnatal transmission.

	Positive breast milk samples	Neonates with COVID‐19 exposed to breast milk
A living systematic review^41^	9/68	4
A systematic review^42^	/	22/190
Observational study^43^	1/14	4/14 (including the one who received mother's milk positive for SARS‐CoV‐2)

### 3.1 Intrauterine transmission: Transplacental transmission

SARS‐CoV‐2 is a large, lipid‐enveloped, single‐stranded RNA virus. It infects host respiratory epithelial cells through ACE2. The placental syncytiotrophoblast, cytotrophoblasts, and the extra‐villous trophoblasts express the ACE2 receptors. Some previous studies have showed the existence of SARS‐CoV‐2 immunoglobulin M/G in neonates born to mothers who are diagnosed with COVID‐19 during pregnancy.^23^, ^24^ Another case report presents findings of a symptomatic individual diagnose with SARS‐CoV‐2 in the third trimester of pregnancy and infant delivered by cesarean section. The mother performed SARS‐CoV‐2 antigen test at the onset of 3 days, result turning to be positive. The neonate delivered by caesarean was negative for SARS‐CoV‐2 nasal swab antigen test 24 h after birth and positive 48 h after birth, and positive for reverse transcription‐polymerase chain reaction (RT‐PCR) test on a nasopharyngeal swab collected 72 h after birth.^25^ Also the presence of SARS‐CoV‐2 or its RNA in placental membrane in some case reports raised the possibility of intrauterine transmission of the virus in utero.^26^, ^27^ But there seems no relationship between placental transmission and neonatal infection. Finding virus in samples such as placental and amniotic fluids does not always means fetal infection.^28^ In this study,^26^ all the neonates tested negative in the first 5 days of life. Meanwhile, these studies^26^, ^27^ did not report whether the swabbed samples are from maternal or fetal side of the placenta, making it difficult to determine intrauterine transmission. Even IgM is not transferred transplacentally in healthy pregnant women, it can be transferred through mother‐to‐child in severe inflammation of the birth canal or presence of viral cytotoxic effects.^29^ Recently, a case of newborn thrombo‐embolism presents strong evidence of intrauterine transmission of SARS‐CoV‐2.^30^ SARS‐CoV‐2 N‐protein and Spike RBD were detected particularly in the esophagus, stomach, spleen, and heart, with a significantly higher H‐Score than the placenta, suggesting a possible intrauterine transmission.

As previous research suggests, when infected with SARS‐CoV‐2, placentas from patients were significantly more likely to show abnormal or injured maternal vessels and intervillous thrombi.^13^ The same as placentas, cerebral venous can also present thrombosis. There was one case that may support the intrauterine mother‐to‐child transmission. A neonate with CVST (cerebral venous sinus thrombosis) was born to an unvaccinated mother with a history of COVID‐19 infection in the beginning of the third trimester. There was normal APGAR score and no history of traumatic birth. The neonate presents fever on the second day of his life and diagnosed with CVST by MRI, but the neonate was negative for nasopharyngeal swab COVID‐19 RT‐PCR testing.^31^ SARS‐CoV‐2 is not known to cause chronic infection. If the neonate infected with SARS‐CoV‐2 in the beginning of the third trimester, neonatal infection is not likely to be active at delivery. In addition, as Bwire et al.^32^ state in their meta‐analysis, the percentage of RT‐PCR positivity to nasopharyngeal swabs is much lower (69.6%) than that observed for bronchoalveolar lavage fluid (91.8%) or rectal swabs (87.8%), In other words, SARS‐CoV‐2 test at birth may be negative even though the newborn has been infected with it. According to NICHD, definition of definitive placental infection with SARS‐CoV‐2 contains documentation of viral presence, location in the placenta tissues, and replication.^33^ Placental infection does not equate with vertical transmission. It may cause placental damage, which leads to vertical morbidity without infection.

For the pregnant, immunological states change with the growth of fetus at different gestational stage: from a pro‐inflammatory state (beneficial to implantation and placentation) to an anti‐inflammatory state (helpful for the fetal growth) in the second trimester, and finally switching to a second pro‐inflammatory state (necessary for the labor) in the third trimester.^34^ So SARS‐CoV‐2 intrauterine transmission may be related to the time when the pregnant women become infected by it.

### 3.2 Contact transmission during delivery: Contact with contaminated amniotic fluid and vaginal secretions

If this transmission exists, it may occur more frequently in natural labor. Neonates would contact with contaminated amniotic fluid and vaginal secretions during delivery. SARS‐CoV‐2 has been detected in the vaginal secretions of COVID‐19‐positive pregnant women, but the detection of the virus in the vaginal secretions may not be associated with neonatal infection.^35^ In this research, only one newborn of COVID‐19‐positive pregnant woman with COVID‐19‐positive PCR, while the test for COVID‐19 in amniotic fluid, vaginal secretions and umbilical blood collected from this pregnant woman were negative. But SARS‐CoV‐2 is not always detected in vaginal secretions. Vaginal swabs during pregnancy and at birth as well as placental ones were negative in more than 98% of women who were SARS‐CoV‐2 positive.^36^ In other studies,^37^, ^38^ all the samples collected from COVID‐19‐positive pregnant women, such as vaginal secretions tested for SARS‐CoV‐2 by RT‐PCR, were negative. Whether the test for vaginal secretions is positive or negative maybe related to the time of maternal infection with SARS‐CoV‐2 and the incubation of virus.

An earlier study suggested that there was no increased risk of infection with SARS‐CoV‐2 for the neonate when birth occurs vaginally.^18^ But now some researches suggest a potentially significant increase in operative delivery among women infected with SARS‐CoV‐2.^39^, ^40^ The reasons for this practice are unclear, but it may be attributable to more aggressive management of labor and delivery during the onset of the pandemic.

### 3.3 Postnatal transmission: By close contact and breast feeding

As we know, the predominant route of SARS‐CoV‐2 transmission is due to close contact by droplet and airborne transmission. So close contact with COVID‐19‐positive mother will greatly increase the risk of SARS‐CoV‐2 infection to neonates. Someone observed that^23^ vertical transmission did occur more frequently among newborns of perinatally symptomatic pregnant people. Another important and possible mother‐to‐child transmission of SARS‐CoV‐2 is breast feeding. Some other virus, such as cytomegalovirus, hepatitis B virus, and human immunodeficiency virus can transmit from women to their babies by breast feeding. Growing evidence has claimed the existence of SARS‐CoV‐2 in breast milk, even it is a rare event in a systematic review,^41^ some breast milk samples were reported to be positive for SARS‐CoV‐2 RNA via RT‐PCR analysis or have specific immunoglobulin G. Another systematic review suggested that the total number of neonates who tested positive in the breastfeeding were significantly higher than that in the bottle‐fed group among mothers diagnosed with SARS‐CoV‐2, even most of mother milk detected no SARS‐CoV‐2 RNA.^42^ In other cases, neonates exposed to maternal breast milk that tested positive for SARS‐CoV‐2 RNA were found to be positive for COVID‐19. But they had been put to skin and roomed‐in without any precautions or stay with other COVID‐19 positive patients in one room.^23^, ^43^ So it is not clear whether the newborn is tested positive due to close contact with confirmed COVID‐19 people or through the exposure to her SARS‐CoV‐2 RNA‐positive breast milk. Since the first wave of the pandemic, several international institutions have issued guidelines that did not recommend the precautionary separation of the infected mother from her child.^44^ Also there is lack of strong evidence that there is increased positive rate among dyads participating in kangaroo care compared to those infants who were not allowed postpartum skin‐to‐skin contact with mothers^42^ Breast feeding and immediate skin‐to‐skin contact are considered standard practice and essential to maternal‐infant bonding, even under the circumstance of COVID‐19 epidemic.

## 4 IMPACT ON NEONATES

Most of the neonates born to infected mothers did not show any clinical abnormalities.^45^ But COVID‐19 infection during pregnancy was associated with substantial risk of morbidity and mortality in postpartum parents and their infants worldwide, compared with their not‐infected pregnant counterparts, especially if these individuals were symptomatic or have comorbidities.^46^ So many researches have revealed that SARS‐CoV‐2 infection during pregnancy was significantly associated with increased risk of adverse maternal outcomes and preterm birth. A multicenter study showed that there were significant associations between prematurity and positive maternal PCR results.^47^ Maternal ICU admission was associated with IUGR, neonatal death, low Apgar score, asphyxia, gestational age, and neonatal weight. Other studies also suggested that preterm birth was the most common compromise for newborns associated with maternal SARS‐CoV‐2 infection, with a rate of proximately 11.1%.^48^, ^49^ In a systematic review, COVID‐19 was not associated with cesarean delivery, but symptomatic COVID‐19 has been associated with higher possibility of C‐section and preterm birth when compared to asymptomatic infection.^50^ The major reasons for preterm delivery include fetal distress and maternal hypoxia.^48^ There are still little infants infected by SARS‐CoV‐2 during perinatal time. Most infected newborns are asymptomatic or mildly symptomatic.^45^ When we diagnose neonates SARS‐CoV‐2 infection, persistence of a pathogen is very important. Persistence of a pathogen, documented through confirmatory testing of a second specimen collected within a few days of the first positive specimen, is critical to be able to distinguish transient colonization, without infection, from true neonatal infection.^51^ Symptoms of COVID‐19 infection occurred more frequently among infants whose mothers were symptomatic compared to those whose mothers were asymptomatic during the perinatal period.^23^ There are also case reports of severe neonatal SARS‐CoV‐2 infection, including cardiorespiratory failure and death.^52^, ^53^ But it is probably related to the gestational age.

Some studies have showed conflict results. A systematic review and meta‐analysis suggested that the COVID‐19 pandemic was associated with a reduction for preterm birth during the pandemic compared with the prepandemic period.^54^ In their opinion, due to COVID‐19 pandemic, there were socioenvironmental and behavioral modifications, such as maternal workload reduction, improved air quality, reduced maternal non‐COVID‐19 related infections, reductions in physical activity, and better nutritional support.^55^, ^56^, ^57^, ^58^ All the measures above are beneficial for the growth of preterm babies. But in this study, women with SARS‐CoV‐2 infection were excluded, it means that the results are not related to the SARS‐CoV‐2 infected pregnant but the general populations. On the other hand, they compared the ratio of preterm babies between pandemic and prepandemic, the definition of compared group was heterogenous. So the result that COVID‐19 pandemic is associated with a reduction for preterm birth should be further verified.

Considering the probably adverse outcomes of SARS‐CoV‐2 infected pregnant women and their babies, protecting the maternal–fetal dyad against COVID‐19 is critical. Vaccination during pregnancy becomes a priority and can generate benefits for both the mother and newborns.^59^ Still, there are few published data on the COVID‐19 vaccine in pregnant women, mainly because they are not usually included in vaccine clinical trials due to safety and responsibility concerns.^60^ Further data is needed to establish knowledge about this condition.

## 5 CONCLUSION

SARS‐CoV‐2 infection in pregnancy can adversely affect the pregnancy and birth outcomes. Especially if these individuals were symptomatic or have comorbidities. Probable vertical transmission route include intrauterine transmission, contact transmission during delivery, and postnatal transmission. Future studies are needed to better understand and identify effective strategies to prevent adverse outcomes in pregnant people with COVID‐19.

## AUTHOR CONTRIBUTIONS

Zhoujie Peng and Wei Xia conceived the study. Wei Xia conducted the online research and wrote the paper. Zhoujie Peng reviewed the manuscript.

## CONFLICT OF INTEREST STATEMENT

The authors declare no conflict of interest.

## ETHICS STATEMENT

Not applicable.

## Data Availability

Data sharing not applicable to this article as no datasets were generated or analyzed during the current study.
